# The Histone Deacetylase Inhibitor (MS-275) Promotes Differentiation of Human Dental Pulp Stem Cells into Odontoblast-Like Cells Independent of the MAPK Signaling System

**DOI:** 10.3390/ijms21165771

**Published:** 2020-08-11

**Authors:** Eun-Cheol Lee, Yu-Mi Kim, Han-Moi Lim, Ga-Eun Ki, Young-Kwon Seo

**Affiliations:** Department of Medical Biotechnology (BK21 Plus team), Dongguk University, Goyang-si 10326, Gyeonggi-do, Korea; eunchul90@gmail.com (E.-C.L.); kjmtik@nate.com (Y.-M.K.); gksahl321@gmail.com (H.-M.L.); gigaeun@naver.com (G.-E.K.)

**Keywords:** epigenetics, dental pulp stem cells, odontoblast, MAPK signaling, cell cycle

## Abstract

The role of dental pulp stem cells (DPSCs) in dental tissue regeneration is gaining attention because DPSCs can differentiate into odontoblasts and other specialized cell types. Epigenetic modification has been found to play an important role in cell differentiation and regulation, among which histone deacetylase (HDAC) is involved in suppressing genes by removing histone acetyl groups. The use of HDAC inhibitor to control this is increasing and has been widely studied by many researchers. This study aimed to induce differentiation by causing epigenetic changes in odontoblast-related genes and the MAPK signaling pathway in human dental pulp stem cells. Western blot and immunofluorescence staining showed increased expression of DMP-1, ALP, DSPP, and RUNX2 compared to the control. However, activation of the MAPK signaling system was similar to but slightly different from the expression of odontoblast-related proteins. After 3 days, as shown by MTT and LDH assays, proliferation decreased overall, but cytotoxicity decreased at only a specific concentration. We confirmed that there was no change in mRNA expression of caspase 3 or 9 using real-time PCR. In addition, flow cytometry analysis confirmed that differentiation occurred due to the decrease in the expression of the CD73 and CD146. Although overall proliferation was reduced due to the G2/M inhibition of the cell cycle, the expression of BCL-2 protected the cells from cell death. Overall, cell proliferation decreased in response to MS-275, but it did not induce cytotoxicity in 5 nM and 10 nM concentration and induces differentiation into odontoblast-like cells.

## 1. Introduction

Dental pulp stem cells (DPSCs) can be obtained from the third molars in a non-invasive way and differentiate into odontoblasts for dentin formation. However, the exact mechanism for the differentiation of DPSCs odontoblast is still unclear.

Epigenetics is the study of the genetic changes in gene expression that do not affect the DNA sequence, affecting how cells read genes. Epigenetic changes are influenced by several factors, including age, environment, and disease state. Mechanisms that have been identified so far include at least four systems: chromatin remodeling; DNA methylation; histone modification; and non-coding RNA expression. They are considered to initiate and maintain epigenetic changes. Among the epigenetic mechanisms mentioned, histone modification by histone acetyltransferases (HATs) and histone deacetylases (HDACs) is important role in regulating cell function [1,2,3]. The HATs/HDACs exhibit different histone substrate binding and catalytic mechanisms. In general, HAT helps promote gene expression by moving acetyl groups to lysine residues in the histone tail, but HDAC suppresses gene expression by removing acetyl groups causing histone hypoacetylation [3,4].

In humans, 18 HDAC enzymes (Class I, II, III, IV) were identified and classified. Among them, Class I, II;, and IV are Zn^2+^-dependent, and Class III; nicotinamide adenin dinucleotide (NAD^+^)-dependent [5,6]. Some of the HDAC inhibitors bind to zinc ions at the catalytic site, blocking Zn^2+^-dependent HDAC enzyme activity, blocking substrate access to the site [7]. As a result, protein accumulation of acetylated histones and other non-histones can progress, affecting cell function and gene expression. Also, HDAC inhibitors are being applied as anticancer agents against several solid tumors, because they enhance the expression of differentiation markers and block the cell cycle in the G2/M phase [4,8,9,10]. An example of an anticancer drug is vorinostat, the first HDAC inhibitor on the market, approved by the FDA to treat cutaneous T cell lymphoma (CTCL) [11]. In addition to vorinostat, some inhibitory compounds have entered clinical trials, such as trichostatin A (TSA), valproic acid (VPA), phenylbutyrate, depsipeptide, pyroxamide, and sodium butyrate (NaB) [4,9,12,13]. The MS-275 (entinostat, SNDX-275) used in this study is also undergoing phase I and phase II clinical trials to suppress HDAC-treated leukemia, lymphoma, or solid tumor patients [14,15,16,17].

The HDAC assay method revealed that even if Zn^2+^-dependent HDAC is inhibited, the targeted HDAC is different for each inhibitor. For example, in the case of MS-275, HDAC 1 and 3 corresponding to class I were selectively suppressed, and class II or IV was not suppressed. In the case of vorinostat, classes I and II were suppressed as a whole, but class IV was not suppressed [18,19]. Also, the concentration used to inhibit HDAC was different for each inhibitor type. Therefore, the application range has recently been widened, and it has been reported that HDAC inhibitors can also be applied to stem cells. At this time, the concentration of HDAC inhibitors applied to stem cells was much lower than the concentration necessary for use as an anticancer drug. There are prior studies of the applications of HDAC inhibitors on stem cells such as inducing osteoblast gene expression [4,11,20], the development of skeletal myocytes [21], and hepatic differentiation [22]. What these previous studies have in common is that they showed the differentiation effects in stem cells in response to HDAC inhibitors. In addition, this epigenetic approach has the potential to treat certain genetic disorders such as cleidocranial dysplasia caused by mutations in Runx2 [23].

Just as tideglusib, which is being developed as a treatment for Alzheimer’s syndrome, showed efficacy in tooth regeneration as a side effect [24], the results of the current study suggest that HDAC inhibitors can be applied to stem cells as well as being used as anticancer drugs. It also supports the theory that inhibiting class 1 HDAC in normal stem cells can induce bone or tooth differentiation. We revealed changes in cell cycles and some other mechanisms in response to HDAC inhibitors.

## 2. Result

### 2.1. Morphology of hDPSCs and Cytotoxic Effect of MS-275

**Figure 1:**
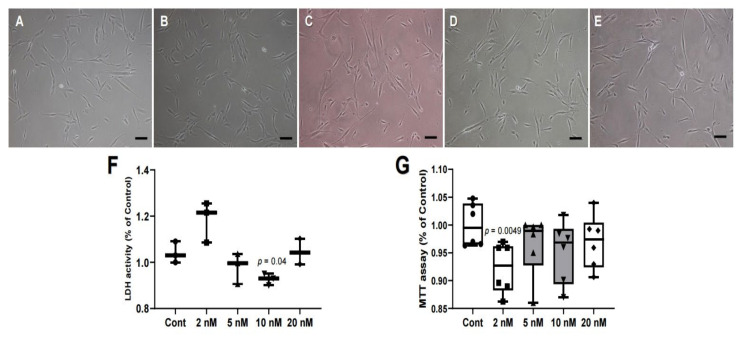
Effect of various concentration of MS-275 on human dental pulp stem cells (hDPSCs) and cell morphology. The hDPSCs (3 × 10^3^/well) were cultured in 3 days. (**A**) Control; (**B**) 2 nM; (**C**) 5 nM; (**D**) 10 nM; (**E**) 20 nM; (**F**) cytotoxic effect of MS-275 by using an LDH assay kit; (**G**) after treatment with MS-275, MTT assay was performed to measure the viability of hDPSCs. Original magnification was 100×; bar = 100 μm. LDH activity: *N* = 3 per treatment. Statistical analysis was performed by one-way ANOVA. MTT assay: *N* = 6. Statistical analysis was performed by student’s *t*-test.

The hDPSCs treated with various concentrations of MS-275 were incubated for 3 days and compared to the MS-275 untreated cells. When comparing the control group and the MS-275-treated group, necrosis and morphological features of apoptosis were not observed after exposure to MS-275. Next, the cellular mitochondrial activity of the hDPSCs was measured by MTT assays and cytotoxicity was measured by LDH assays. The treated cells had similar proliferation and cytotoxicity levels as the control. Therefore, no cytotoxicity was observed when cells were exposed to MS-275 at concentrations below 20 nM (Figure 1).

### 2.2. Western Blotting

**Figure 2:**
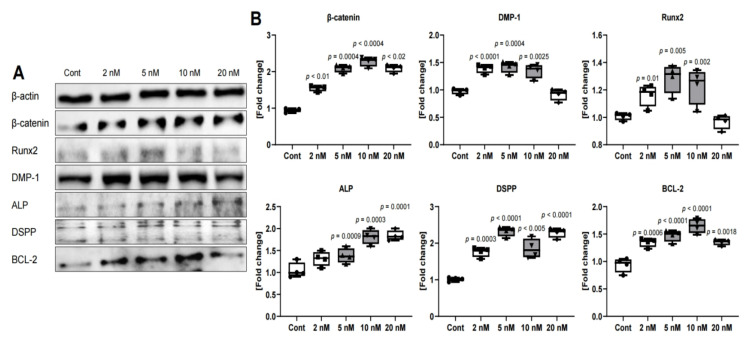
Western blot analysis of protein expression in human dental pulp stem cells (hDPSCs) treated with an MS-275 in 3 days. (**A**) Western blot image of β-catenin, Runt-related transcription factor 2 (Runx2), Dentin matrix acidic phosphoprotein 1 (DMP-1), Alkaline phosphatase (ALP), Dentin sialophosphoprotein (DSPP), and B-cell lymphoma 2 (BCL-2) using β-actin as a control; (**B**) protein expression level of β-catenin, Runx2, DMP-1, ALP, DSPP, and BCL-2 (with the control as the standard). β-Actin was used in each lane as an internal control. *N* = 4. Statistical analysis was performed by one-way ANOVA and student’s *t*-test.

When exposed to MS-275 during the culture period, dentin-related proteins (Runx2, DMP-1, ALP, and DSPP) were upregulated at all concentrations as compared to the control (untreated cells). Among them, the expression was more than 1.6 fold higher on average in the 5 nM and 10 nM groups. The expression of BCL-2, an anti-apoptosis protein, was also increased by more than 1.4 fold at 5 nM and 10 nM (Figure 2). 

**Figure 3:**
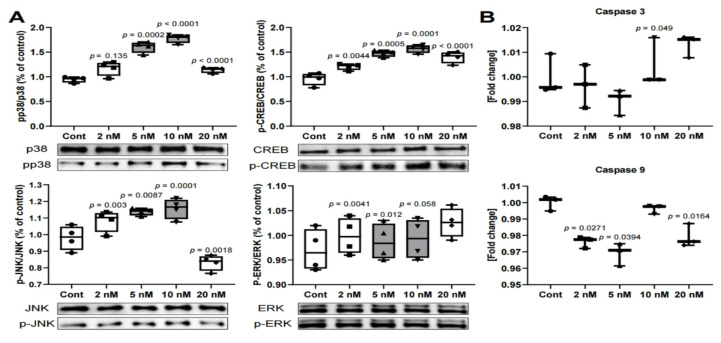
(**A**) After exposure to MS-275, phosphorylated p38, c-Jun N-terminal kinase (JNK), and cAMP response element binding (CREB) for protein expression levels were analyzed using Western blot; (**B**) effect of MS-275 on caspase 3 and caspase 9 gene expressions in hDPSCs after 3 days culture. β-Actin was used as an internal control for mRNA. Western blot: *N* = 4. RT-PCR: *N* = 3. Statistical analysis was performed by one-way ANOVA and student’s *t*-test.

Next, we found that MS-275 induced a significant increase in p38/JNK/CREB phosphorylation and β-catenin expression. The β-catenin levels markedly increased by 2.0 fold after treatment with 5 nM or 10 nM of MS-275. CREB phosphorylation and p38 phosphorylation were markedly increased by approximately 1.6 fold. In addition, JNK phosphorylation was also increased by 1.2 fold compared to the control. However, ERK phosphorylation did not show any difference from the control (Figure 3A). Therefore, when exposed to MS-275, odontoblast differentiation generally occurs, but differentiation proceeds more stably as p38, JNK, CREB, and β-catenin are activated at specific concentrations (5 nM, 10 nM). 

### 2.3. Quantitative Real-Time Polymerase Chain Reaction (RT-PCR)

As shown in Figure 3B, the expression levels of caspase 3 and 9, which play central roles in cell death, showed similar results to the control group. These findings are similar to the ERK phosphorylation findings and indicate MS-275 does not induce cytotoxicity at the selected concentrations. They support the findings that the p38 and JNK signaling pathways have a role in helping odontoblast differentiation.

### 2.4. Immunofluorescence Staining Analysis

**Figure 4:**
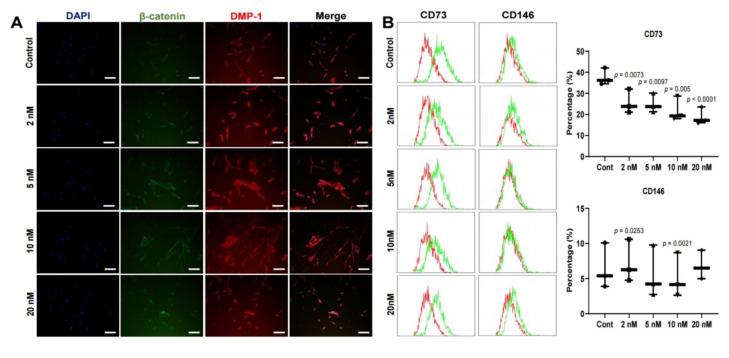
(**A**) Immunofluorescence staining of human dental pulp stem cells (hDPSCs) after treatment with MS-275; immunostaining with β-catenin and DMP-1 antibody. Original magnification was ×100; bar = 100 μm; (**B**) Flow cytometry of cluster of differentiation (CD) marker in hDPSCs treated with an MS-275 in 5 days. CD73 and CD146 were used as a positive marker. *N* = 3. Statistical analysis was performed by one-way ANOVA and student’s *t*-test.

β-Catenin, a gene transcription marker, and DMP-1, a marker of mineralization of bone and dentin, were highly expressed at 5 nM and 10 nM. These results indicate the induction of odontoblast differentiation at specific concentrations as evidenced by the Western blot (Figure 4A).

### 2.5. Flow Cytometry

Flow cytometry confirmed that the expression level of CD73 was 10% lower than that of the control, and CD146 decreased by 2%. This is similar to Western blot results with increased odontoblast-related protein at concentrations of 5 nM and 10 nM and immunofluorescence results with increased expression of b-catenin and DMP-1. As a result, it is suggested that the expression level of positive markers, which indicate the activity of these stem cells, was reduced because differentiation was induced. The expression levels of both positive and cell surface markers were not significantly (* *p* < 0.05) different among three of DPSC lines (Figure 4B).

**Figure 5:**
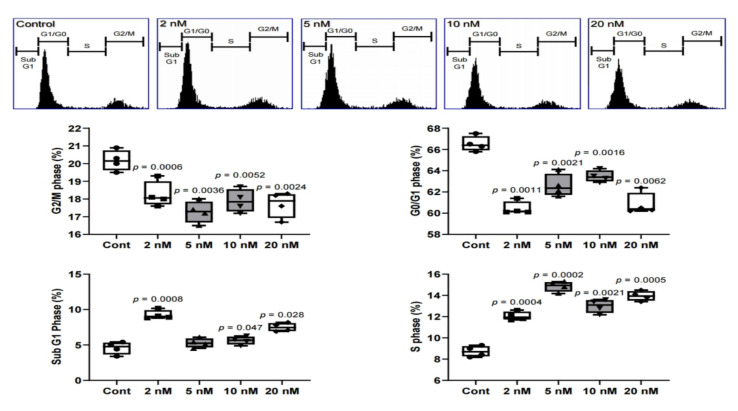
Propidium iodide (PI) staining in human dental pulp stem cells (hDPSCs) treated with an MS-275 in 5 days. *N* = 4. Statistical analysis was performed by one-way ANOVA and student’s *t*-test.

In the PI staining results, sub G1, which means apoptosis, showed similar levels to the control at 5 nM and 10 nM. In addition, the overall proliferation decreased when the cells were exposed to MS-275. The G0/G1, which means the resting period, had the highest proportion at 5 nM and 10 nM. Based on the Western blot, fluorescence, and CD marker results, it can be suggested that differentiation was induced at 5 nM and 10 nM. On the other hand, the control group was observed to be in the proliferative phase. In fact, the area of G2/M was the highest, 20.3%, for the control. Therefore, 5 nM and 10 nM of MS-275 did not cause apoptosis and instead triggered differentiation into odontoblasts (Figure 5).

## 3. Discussion

Histone deacetylase (HDAC) inhibitors are promising anticancer drugs due to the fact of their ability to promote terminal differentiation and/or induce growth arrest of numerous types of tumor cells [4]. HDAC inhibitors have also been applied to the treatment of genetic diseases such as increased acetylation of Runx2 in a calvaria defect model of the Runx2 null mouse [23] and improving mRNA splicing to treat spinal muscular atrophy [5]. The reason for this diversity in applications is that HDAC-related genes are different. Therefore, the gene affected depends on the type of HDAC inhibitor [3].

HDAC is classified as Zn^2+^-dependent (class I;, class II, class IV) and nicotinamide adenin dinucleotide (NAD^+^)-dependent (Class III: sirtuins) [6,25]. The Zn^2+^-dependent members can remove acetyl groups from histone to close the space between histones and DNA, thereby controlling cell survival, proliferation, and gene regulation [26,27]. According to a recent report, class I (HDAC 1, 2, 3) were downregulated during osteogenic differentiation in human PDLCs. At this time, the HDAC inhibitor was used to effectively increase the acetylation of H3K9K14 (Histone H3, Lysine 9, 14) and increase the expression of bone-related genes [28]. Since HDAC class I is mainly located in the nucleus and ubiquitously rather than in specific tissues [6], there are reports that it has promoted the differentiation of osteogenesis and odontoblast in various cells such as adipose-derived stromal cells, bone marrow [29], umbilical cord [8], and dental pulp stem cells [30].

The inhibitors are classified into hydroxamates, aliphatic acids, benzamides, and cyclic peptides to their structure [25]. The MS-275 used in this study belongs to benzamides. They inhibit activity by substituting zinc ions in the HDAC structure. By inhibiting HDAC, it is possible to induce the accumulation of proteins of acetylated histones and other non-histones, thereby regulating cell function and gene expression [3,26,27,28]. Therefore, this study supports prior studies using HDAC inhibitors to promote regeneration in dental pulp, suggesting that MS-275 can also play a role. The low concentration of MS-275 used did not significantly affect the proliferation of DPSC compared to the control group, but the proliferation of DPSC treated with MS-275 showed decreased proliferation at a specific concentration (Figure 1). These results are expected to be due to the upregulation of cellular antioxidant pathways, increased expression of the anti-apoptotic protein BCL-2, the stress-responsive transcription factor NF-κB, and the use of alternative gene silencing pathways such as DNA methylation [31]. In fact, in this experiment, MS-275 significantly increased the expression of BCL-2 compared to the control (Figure 2). In particular, its expression level was the highest at 10 nM.

These results indicate that MS-275 has a limited useful concentration range, and apoptosis or necrosis may occur if concentrations exceeding that limit are used. In fact, apoptosis and necrosis occurred at concentrations exceeding 20 nM (data not shown). The drug resistance to BCL-2 has been reported in previous studies of chronic lymphocytic leukemia cells. Here, MS-275 showed high expression levels of BCL-2 protein at 300 nM and 3 μM, but its expression decreased with increasing exposure times and concentrations. As a result, the expression level of caspase increased causing apoptosis [32].

Caspase 3 is an essential component of extrinsic and intrinsic apoptosis, and caspase 9 activates caspase 3 [33]. Caspase 3 relies on the MAPK signaling system to mediate its effects [34,35]. Among them, the ERK signaling pathway is involved in both physiological and pathological cell proliferation and suppressing ERK1/2 is a natural way to treat cancer cells [36]. Because of this, the use of HDAC inhibitors may provide a basis for the development of a wide range of cancer treatments in which the ERK pathway is continuously activated [37]. JNK and p38 also regulate apoptosis, but they are more sensitive to toxic reactions due to the external stimuli, including drugs, than ERK [38,39].

Previous studies on apoptosis in odontoblast affected by sodium fluoride (NaF) showed that caspase has a strong dependence on JNK [34,35]. Similarly, in that study, as evidence of drug resistance, JNK and p38 were activated, and the anti-apoptosis protein BCL-2 was overexpressed (Figure 2 and Figure 3A). However, the authors of that study used a much higher concentration of HDAC inhibitor than in this experiment, and the results of this study showed similar expression levels of caspase 3 and 9 in both the treatment and control groups (Figure 3B). The ERK phosphorylation also showed a similar level in both groups. As a result, no significant cytotoxicity was observed below 20 nM, suggesting that it was a suitable concentration that could only act as a minimal defense against MS-275. These findings suggest that the role of MAPKs in MS-275’s effects may vary according to cell types.

The odontoblast-related proteins induced by MS-275 were similar to the signaling pathway of MAPK but showed different results. These results suggest that HDAC inhibitors are directly involved in histones and the regulation of gene expression regardless of the external signaling systems [2,3]. When exposed to MS-275, the overall expression level was higher than that of the control group, but it showed the highest expression levels at 5 nM and 10 nM, showing a differentiated result.

This differentiated result was also observed in the results of fluorescence staining and flow cytometry analysis, where CD73 and CD146 are positive cell surface CD markers strongly expressed in MSCs. In this study, CD73 was decreased by more than 10% compared to the control, and CD146 was also decreased. These results suggested that differentiation was induced by MS-275 (Figure 4). The results of Western blot and fluorescence staining also suggested that MS-275 promotes cell differentiation, since the expression of odontoblast-related proteins was significantly lower in the control group.

A small change occurred in the cell cycle, detected by PI staining. The area of sub G1, which indicates apoptosis, increased except for the 5 nM and 10 nM groups as compared to the control (Figure 5). These results are consistent with the results of the proliferation and cytotoxicity assays. Cytotoxicity increased except for at 5 nM and 10 nM, and proliferation decreased at all concentrations compared to control (Figure 1). This suggests that HDAC inhibitors affect the cell cycle and reduce proliferation by arresting the cells in G2/M [8]. This experiment suggests that the effect is applied not only to cancer cells but also to stem cells.

**Figure 6:**
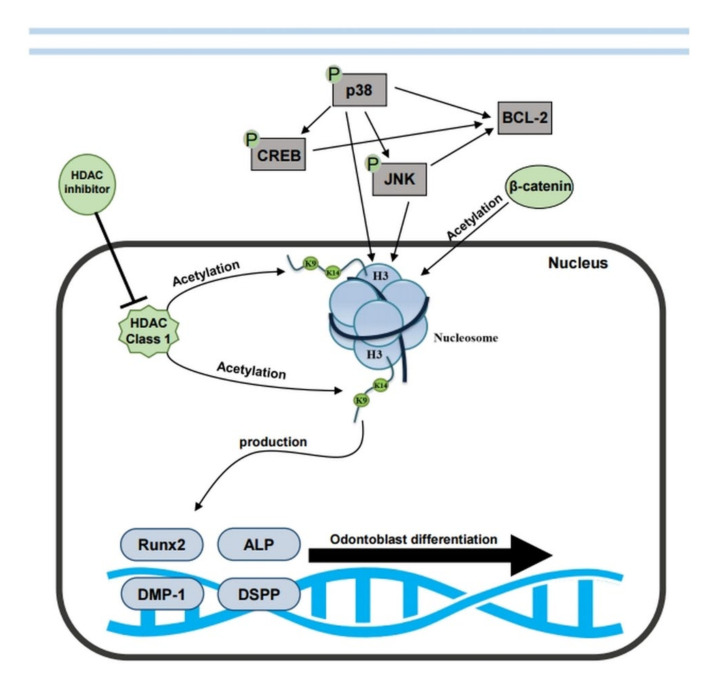
Mechanism of HDAC inhibitor MS-275 in odontoblast differentiation of hDPSCs. MS-275 inhibits HDAC enzymes including the production and acetylation of Runx2, ALP, DMP-1, and DSPP. As a result, it reduces Class 1 HDACs and activates dentin-related gene expression. Cells then go to odontoblast differentiation. (H3: Histone H3; K9, K14: Lysine 9, 14).

In summary, DPSCs exposed to MS-275 reduced cell proliferation due to the arrest in G2/M, and there was increased expression of BCL-2 protein mediated by p38 and JNK to reduce cytotoxicity. At the same time, by inhibiting the class I HDACs, the expression of odontoblast-related genes improved, and this process was performed independently of the MAPK signaling pathway. Overall, the results of flow cytometry suggested that differentiation occurred in response to specific concentrations of MS-275. However, further analysis with the MAPK signaling system suppressed is necessary. Finally, HDAC inhibitors can directly enter the nucleus of cells and regulate gene expression without the need for an external signaling system, and their effect is much faster and more potent (Figure 6). In addition, our results suggest that the concentration of HDAC inhibitors required for anti-proliferation for cancer cells and the concentration necessary for differentiation of stem cells are different, and when appropriate concentrations are applied to stem cells, they do not cause cytotoxicity [1].

## 4. Materials and Methods

### 4.1. Cell Culture

Human dental pulp stem cells (hDPSCs, Lonza, PT-5025, Basel, swiss) were cultured in M-DMEM supplemented with 10% fetal bovine serum, 100 U/mL penicillin G, and 100 μg/mL streptomycin at 37 °C in a 5% CO_2_ incubator. For the experiments, hDPSCs were cultured in standard odontogenic induction medium, namely, M-DMEM supplemented with 10% FBS, antibiotics, 10 nM dexamethasone (Sigma–Aldrich, D4902, St Louis, MO, USA), 50 ng/mL BMP-2 (Sigma–Aldrich, B3555, St Louis, MO, USA), 20 ng/mL TGF-β1 (Bio Legend, 580704, San Diego, CA, USA), and 5 ng/mL FGF-2 (Bio Legend, 571504, San Diego, CA, USA) for 3 or 5 days at 37 °C in a 5% CO_2_ incubator. For drug exposure, MS-275 (Apexbio, A8171, Houston, TX, USA) was initially dissolved in dimethyl sulfoxide (DMSO), then the concentration was adjusted with PBS, and it was added to the media. The medium was changed every 2–3 days. The hDPSCs from passages 3 to 5 were used for the experiments. DMSO (Sigma-Aldrich, D5879, St Louis, MO, USA) was added to the control at an additional 1/5000 to offset the effect of DMSO on the experiment.

### 4.2. Cell Proliferation Assay

Cells were seeded at 3 × 10^3^ cells per well in 12 well plates and cultured with odontoblast media with MS-275 for 3 days. For the 3-(4,5-dimethylthiazol-2-yl)-2,5-diphenyltetrazolium bromide (MTT) assays, each well was treated with 500 uL of MTT solution (3 mg/mL in PBS, Sigma–Aldrich, M5655, St Louis, MO, USA), and the plates were incubated at 37 °C for 1 h. Subsequently, formazan was dissolved by replacement with DMSO and mixed for 5 min. Absorbance was measured at 570 nm with the ELISA plate reader (Molecular Devices, VersaMax, San Jose, CA, USA).

### 4.3. Cytotoxicity Assay

The LDH-LQ kit (Takara, MK401, Seoul, Korea) was used to evaluate cytotoxicity. Briefly, the medium of the cultured cells for 3 days was aliquoted (100 μL), transferred to a 96 well plate, and 50 μL of a working solution was added, followed by incubation at room temperature for 30 minutes. The reaction was terminated by adding 50 μL 1 N HCl, and the absorbance was measured at 490 nm with an ELISA plate reader (Molecular Devices, VersaMax, San Jose, CA, USA).

### 4.4. Western Blotting

Cells were seeded at 0.7 × 10^5^ cells per well in 100 mm dishes and cultured with odontoblast media with MS-275 for 3 days. Then, the cells were lysed in sample buffer consisting of 4% sodium dodecyl sulfate (SDS), 5% 2-mercaptoethanol, 40% glycerol, 0.05% bromophenol blue in Tris-HCl, pH 6.8, and it was boiled at 100 °C for 10 min. The total protein concentration was measured with a BCA assay. After the sample was treated with SDS-page, the protein in the gel was transferred to a nitrocellulose membrane. Subsequently, membranes were incubated with the primary antibodies: anti-β-actin (Abcam, ab8227, Cambridge, MA, USA), anti-β-catenin (Santa Cruz, sc-7963, Santa Cruz, CA, USA), anti-RUNX2 (Cell Signaling, 12556, Danvers, MA, USA), anti-DMP-1 (Abcam, ab103203), anti-ALP (Abcam, ab75699), anti-BCL-2 (Abcam, ab32124, Cambridge, MA, USA), anti-DSPP (Santa Cruz, sc-73632, Santa Cruz), anti-p38 (Antibodies Online, ABIN2957701, Aachen, Germany), anti-p-p38 (Thermo Fisher Scientific, MA5-15177, Waltham, MA, USA), anti-JNK (Abcam, ab208035, Cambridge, MA, USA), anti-p-JNK (Abcam, ab76572, Cambridge, MA, USA), anti-ERK (Cell Signaling, 4695, Danvers, MA, USA), anti-p-ERK (Cell Signaling, 4370, Danvers, MA, USA), anti-CREB (Cell Signaling, 9197, Danvers, MA, USA), and anti-p-CREB (Cell Signaling, 9198, Danvers, MA, USA). Membranes were then washed four times using TBS-T (0.1% Tween in TBS) and further incubated with the secondary antibody: anti-rabbit (Abcam, ab6721, Cambridge, MA, USA) or anti-mouse (Abcam, ab6728, Cambridge, MA, USA) for 2 h at room temperature. Protein detection was performed using a Lumi Femto (Daeil Laboratory Service, DG-WF100, Seoul, Korea) and Molecular Imager ChemiDoc XRS+ station (Bio-Rad, Hercules, CA, USA). Western blot images were analyzed and quantified through ImageJ software (National Institutes of Health).

### 4.5. Quantitative Real-Time Polymerase Chain Reaction (RT-PCR)

To quantify the relative gene expression levels, total RNA was extracted from each sample using 500 μL of TRIzol (Invitrogen, 15596026, Waltham, MA, USA). Then, we added 100 μL of chloroform (Merck, 288306, Darmstadt, Germany). After centrifugation (12,000 rpm, 4 °C, 15 min), the upper portion was transferred to new e-tubes, and 500 μL of isopropanol was added. After incubation at room temperature for 10 minutes and further centrifugation (14,000 rpm, 4 °C, 10 min), the upper phase was discarded. The pellet was washed with 1 mL of 70% ethanol and centrifuged (9500 rpm, 4 °C, 5 min). The upper phase was removed, and the pellet was air-dried. The pellet was dissolved in 20 μL of DEPC-treated water and incubated for 10 min on ice. Total RNA concentration was measured using a Nano Drop device (BioTek, Cytation 3 imaging reader, Winooski, VT, USA). The cDNA was synthesized from 2 μg of total RNA following the protocol using the Advantage RT-PCR kit (Takara, 639543, Seoul, Korea). The gene expression levels of the target genes caspase 3 and caspase 9 were measured by using a StepOnePlus Real-Time PCR System (Thermo Fisher Scientific, 4376592, Waltham, MA, USA). The following primer sequences were used: Caspase 3-Forward, 5′-GGA AGC GAA TCA ATG GAC TCT GG-3’, and Caspase 3-Reverse, 5′-GCA TCG ACA TCT GTA CCA GAC C-3′; Caspase 9-Forward, 5′-GTT TGA GGA CCT TCG ACC AGC T-3′, and Caspase 9-Reverse, 5′-CAA CGT ACC AGG AGC CAC TCT T-3′.

### 4.6. Immunofluorescence Staining Analysis

The cells were fixed for 10 min at room temperature using 4% paraformaldehyde prepared in PBS. Cells were immunostained according to standard protocols, using the following primary antibodies: anti-β-catenin (Santa Cruz, sc-7963, Santa Cruz, CA, USA) and anti-DMP-1 (Abcam, ab103203, Cambridge, MA, USA), and the appropriate fluorescent secondary antibodies: anti-rabbit (Cell Signaling, 4413) or anti-mouse (Cell Signaling, 4408, Danvers, MA, USA). Representative images were captured by using a Nikon Eclipse Ti microscope, and analyzed the photos through ImageJ software (National Institutes of Health).

### 4.7. Flow Cytometry

The cells (0.5 × 10^5^/well) were seeded in 100 mm culture dishes and allowed to attach for 24 h. The cells were treated with the indicated drugs for 5 days and harvested after Accutase (Innovative Cell Technologies, CA, USA). Markers included antibodies targeting CD73 (Bio Legend, 344003, San Diego, CA, USA) and CD146 (Bio Legend, 361005) as positive stem cell markers. As controls, the cells were incubated with mouse isotype controls (PE-IgG, Bio Legend, 409303). Fluorescence of the stained cells was captured in an Accuri C6 (BD Biosciences, CA, USA), and after the acquisition of 100,000 events, the data were analyzed using FCS Express software. The data are expressed as the percentage of cells positive for each marker.

For cell cycle analysis, collected cells were washed with PBS and then fixed with ethanol at 4 °C for 30 min. Fixed cells were washed with PBS two times and incubated with RNase A (10 mg/mL, Thermo Scientific, EN0531) at room temperature for 5 min. The cells were stained with propidium iodide (1 mg/mL, Thermo Scientific, P3566, Waltham, MA, USA) for 5 min in the dark. Cell cycle analysis was performed using an Accuri C6 (BD Biosciences, CA, USA).

### 4.8. Statistical Analysis

Statistical analyses were performed using Graphpad Prism 8.4.2 software. All data are visualized using box and whisker plots with the median represented by the line. Data were analyzed by one-way analysis of variance (ANOVA). For comparisons between two groups, the Student’s *t*-tests was used. Each data point represents the average standard error of three or more independent experiments (*n* ≥ 3). A *p* < 0.05 or < 0.01 was considered statistically significant.

## 5. Conclusions

Class 1 HDAC inhibitors have the potential to treat damaged dentin by inducing differentiation of DPSC into odontoblast-like cells. However, there is a need to verify the efficacy of dentin regeneration through further in vivo experiments.
